# Prevention of Post-Operative Adhesions: A Comprehensive Review of Present and Emerging Strategies

**DOI:** 10.3390/biom11071027

**Published:** 2021-07-14

**Authors:** Ali Fatehi Hassanabad, Anna N. Zarzycki, Kristina Jeon, Jameson A. Dundas, Vishnu Vasanthan, Justin F. Deniset, Paul W. M. Fedak

**Affiliations:** 1 Section of Cardiac Surgery, Department of Cardiac Sciences, Libin Cardiovascular Institute, Cumming School of Medicine, University of Calgary, Calgary, AB T2N 2N9, Canada; ali.fatehihassanabad@ahs.ca (A.F.H.); annazarzycki00@gmail.com (A.N.Z.); jameson.dundas@ucalgary.ca (J.A.D.); vishnu.vasanthan@ucalgary.ca (V.V.); jdeniset@ucalgary.ca (J.F.D.); 2 Department of Anesthesiology and Pain Medicine, Faculty of Medicine and Dentistry, University of Alberta, Edmonton, AB T6G 2R7, Canada; kjeon@ualberta.ca; 3 Department of Physiology and Pharmacology, University of Calgary, Calgary, AB T2N 1N4, Canada

**Keywords:** post-surgical adhesions, immune mediators, pharmaceuticals, barriers, biomaterials

## Abstract

Post-operative adhesions affect patients undergoing all types of surgeries. They are associated with serious complications, including higher risk of morbidity and mortality. Given increased hospitalization, longer operative times, and longer length of hospital stay, post-surgical adhesions also pose a great financial burden. Although our knowledge of some of the underlying mechanisms driving adhesion formation has significantly improved over the past two decades, literature has yet to fully explain the pathogenesis and etiology of post-surgical adhesions. As a result, finding an ideal preventative strategy and leveraging appropriate tissue engineering strategies has proven to be difficult. Different products have been developed and enjoyed various levels of success along the translational tissue engineering research spectrum, but their clinical translation has been limited. Herein, we comprehensively review the agents and products that have been developed to mitigate post-operative adhesion formation. We also assess emerging strategies that aid in facilitating precision and personalized medicine to improve outcomes for patients and our healthcare system.

## 1. Introduction

Surgical adhesions are pathological fibrotic connections that form between organ surfaces and the walls of surrounding body cavities following tissue trauma and ischemia. Post-surgical adhesions can range from thin films of connective tissue to thick fibrous bridges that are vascularized and innervated [1]. Despite improvements in surgical technique, adhesions are a common post-surgical complication, developing after 50–95% of all operations regardless of procedure or anatomical location [2,3,4]. Adhesion formation following pelvic, peritoneal, and thoracic operations have been the focus of much of the current literature [5,6,7,8,9,10,11]. Adhesions represent a significant clinical problem that impacts millions of patients annually [12]. They cause complications such as severe chronic pain, organ dysfunction, and increase the risk of repeat surgeries, including surgeries to address the adhesions themselves. In the operating room, adhesions increase the risk profile at the time of repeat operation due to hemorrhage, perforation, reduced surgical exposure, and prolonged operative times [13,14,15,16,17], while also conferring major financial burden on the healthcare system [18,19,20,21,22].

Although the clinical impact of post-operative adhesions is appreciated, the pathophysiology of adhesions formation and mechanisms underlying their formation are not yet fully established. Studies have suggested that adhesion formation is a dynamic but dysregulated regenerating tissue repair process that can be characterized by distinct cellular and immune responses [23,24,25]. An important feature of tissue repair and potential pathologic adhesion formation is the local inflammatory response, tissue damage, and hypoxia induced by surgical trauma [26,27,28]. Post-surgical adhesion formation involves three core processes: (1) inhibition of the fibrinolytic and extracellular matrix degradation systems, (2) the induction of an inflammatory response involving the production of cytokines and transforming growth factor-β (TGF-β), and (3) induction of tissue hypoxia, leading to increased expression of vascular endothelial growth factor (VEGF) [29]. Factors that have been implicated in post-operative adhesion formation are summarized in Table 1.

Given our poor understanding of the pathophysiology of post-surgical adhesion formation, there is an unmet clinical need for the development of safe and effective therapeutic options that can be used to mitigate them. Different pharmacological strategies have been employed to reduce adhesion formation, severity, and chronicity. Biomaterials have also been designed to act as tissue barriers that can physically isolate wounds and have been shown to prevent the formation of adhesions to varying degrees of success. In an effort to facilitate precision medicine, groups have recently assessed the potential of nanoparticles and gene therapy in preventing post-surgical adhesion formation, although their clinical translation has been limited. Herein, we comprehensively review the major types of established and emerging strategies that have been used to prevent or lessen post-operative adhesion formation in various types of surgeries. We also offer our perspective on how emerging methods can be effectively translated clinically.

## 2. Surgical Approaches and Adhesions

Some groups have hypothesized that minimally invasive surgical approaches may reduce the risk of post-operative adhesion formation. This has been based on the theory that minimally invasive approaches lead to less trauma, which should result in less post-injury repair processes being activated [98,99,100]. However, literature has yet to conclusively prove that this is indeed the case in clinical practice. A systematic review and meta-analysis of different surgical techniques, including laparotomy and laparoscopy, found that various approaches did not reduce post-operative peritoneal adhesions, small bowel obstruction, or infertility rates [101]. In contrast, some preclinical and clinical studies have shown that laparoscopic approaches reduce the rate of post-operative adhesions, especially in the context of adhesiolysis [102,103,104]. It is also important to note that some of the benefits of minimally invasive surgery with respect to post-operative adhesion formation may be limited given the use of pneumoperitoneum with the use of CO2. Pneumoperitoneum is associated with increased intra-abdominal pressure, which may compromise normal blood flow and result in ischemic injury, including acidosis and production of reactive oxygen species (ROS) [30,105,106,107,108,109]. The potential role of temperature and humified gas in post-surgical adhesion formation has also been assessed in pre-clinical studies. Hypothermia with a humidified gas mixture of CO2, N2O, and O2 seems to provide the biggest impact in preventing post-operative intra-abdominal adhesion formation in rodents [110,111]. Ultimately, meticulous and minimal tissue handling, preventing thermal injury, optimal hemostasis, maintaining a moist operative field, reducing the risk of infection, and avoiding the use of foreign body material may be the most important factors in reducing the formation of post-operative adhesions [112].

## 3. Pharmaceutical Strategies for Adhesion Prevention

Different classes of medications and pharmaceuticals have been assessed for their anti-adhesive properties. Groups have investigated these agents based on their effects on various mechanisms that have been implicated in adhesion formation, including the coagulation cascade and different components of inflammatory pathways.

### 3.1. Agents Targeting Angiotensin

The renin-angiotensin-aldosterone system (RAAS) is the principal regulator of blood pressure; recent studies also indicate the vital role of RAAS in inflammation, proliferation, and fibrosis pathways, and particularly in post-surgical adhesions [113,114,115,116,117,118,119]. Renin cleaves angiotensinogen to produce the inactive peptide angiotensin I, which is further cleaved by the angiotensin-converting enzyme (ACE) to produce the active peptide angiotensin II (although there are alternative routes). Most of the known functions of the RAAS are mediated through the activation of the angiotensin II type 1 receptor (AT_1_ receptor) by angiotensin II [120]. Angiotensin, the main peptide of the RAAS, has proinflammatory and pro-fibrotic activity, inducing cell hypertrophy, the expression of extracellular matrix proteins, and promoting TGF-β and other profibrotic molecule signalling [121,122].

Angiotensin type 1 (AT_1_) receptor antagonists are known to reduce plasminogen activator inhibitor-1 (PAI-1) expression, which is a key component of the fibrin production-fibrinolytic balance [123,124,125]. In a rat model of intra-peritoneal adhesions, the administration of candesartan, the AT_1_ receptor agonist, resulted in a marked decrease in the severity of intra-peritoneal adhesions and PAI-1 mRNA expression [126]. No human studies have thus far focused on the use of AT_1_ receptor antagonists for preventing adhesion formation. Compound 21 (C21) is a small molecule angiotensin type 2 (AT_2_) receptor agonist. Contrary to AT_1_ receptor agonists, engagement of AT_2_ receptors by angiotensin II leads to anti-fibrotic effects, particularly the down-regulation of TGF-β [127]. C21 has been shown to reduce fibrosis associated with myocardial infarction, stroke, renal disease, and idiopathic pulmonary fibrosis [128,129,130,131]. C21 is also reported to reduce intra-peritoneal adhesion formation in mice, and has inhibitory effects on mesothelial cell and peritoneal fibroblast migration, TGF-β levels, and pSMAD2/3 expression, all of which have been shown to contribute to adhesion formation [132].

Angiotensin converting inhibitors (ACE-I) have been used to suppress growth factor signalling and attenuate the formation of fibrosis and post-operative adhesions [133]. ACE-I has been suggested to target the epidermal growth factor (EGF) and/or the TGF-β signalling cascades [133]. Administration of high-doses of ACE-I impair post-operative wound and bowel anastomotic healing, while low doses do not [134]. Studies have also demonstrated a decrease in TGF-β-mediated mesothelial fibrosis with ACE-I and angiotensin receptor blockers in vitro [135]. Taken together, these studies indicate that there is a potential benefit in targeting angiotensin receptors and related enzymes for reducing adhesion formation. However, more clinical studies are required to better assess the role these agents can have in the clinical setting.

### 3.2. Hypoxia-Inducible Factors (HIF) Inhibitor and N-Acetyl-Cysteine

Hypoxia has been shown to play a key role in adhesion formation, so studies have investigated whether inhibiting hypoxia-driven pathways is beneficial in mitigating post-surgical adhesion formation [83,136]. Administration of HIF-inhibitor in mice before and after induction of peritoneal adhesions has been associated with a recent study, which showed that disruption of the HIF1-alpha pathway using small molecule inhibitor, YC-1 (3-[5′-Hydroxymethyl-2′-furyl]-1-benzyl-indazole), was sufficient to curtail adhesion formation in a mouse model of peritoneal adhesions [137]. Administration of this HIF-alpha inhibitor was associated with compromised pro-inflammatory activation of macrophages, reduced activation of peritoneal fibroblasts, and enhanced fibrinolysis [137]. With the growth of specific and targeted monoclonal antibodies [138], leveraging the HIF1-alpha pathway for preventing adhesion development has great clinical promise as it will facilitate precise and personalized medicine.

N-acetyl-cysteine (NAC) is a precursor of glutathione and an antioxidant bearing sulfhydryl groups with excellent free radical scavenging. The generation of harmful ROS plays a key role during postoperative adhesion formation following hypoxic conditions. N-acetyl-cysteine has been found to be effective in preventing post-operative pericardial adhesions in a rabbit model when applied with a sponge, which was then removed from the surgical site [139]. The authors postulate that this effect may be largely attributed to the anti-fibrotic capacity of NAC as well as its ability to serve as a physical barrier between serosal surfaces, which is not well explained. Other studies have reported that NAC decreases abdominal adhesion formation in rats via the upregulation of peritoneal fibrinolytic activity [140]. For there to be a meaningful clinical translation and application more in vitro and in vivo studies are warranted.

### 3.3. 3-Hydroxy-3-Methylglutaryl Coenzyme A (HMG-CoA) Reductase Inhibitors

Statins are a class of lipid-lowering medications that inhibit HMG-CoA reductase (HMGCR), the rate-limiting enzyme of the mevalonate pathway. Statins have also been demonstrated to have anti-inflammatory and pro-fibrinolytic capabilities [141], and have anti-adhesive properties within their therapeutic dose range [142]. In a rat model, oral fluvastatin administered after peritoneal surgery significantly reduced adhesions, which was attributed to an upregulation of matrix metalloproteinase (MMP)-9 expression, an important regulator of extracellular matrix production [143]. Additionally, fluvastatin down-regulated the levels of IL-1b, a pro-inflammatory cytokine, and inhibited the invasion of inflammatory cells, resulting in a reduction in fibrous adhesion formation and an increase in the activity of tPA [143]. Similar results have been obtained using lovastatin and atorvastatin, which have been mechanistically identified to increase tPA mRNA levels in peritoneal tissue and tPA activity, leading to the upregulation of local fibrinolysis [144]. More studies are needed to further assess the role this class of medications can have in preventing post-surgical adhesion formation.

### 3.4. Neurokinin-1 Receptor (NK-1R) Antagonist

One of the main ligands of Neurokinin-1 Receptor (NK-1R) is the pro-inflammatory peptide substance P, which is involved in inflammation and wound healing, and may be an important signalling molecule in peritoneal adhesion formation [145]. The application of NK-1R agonists in animal models of post-operative adhesions leads to significant reduction in adhesions, as well as increased activity of MMPs, fibrinolysis, and lower levels of oxidative stress [145,146,147,148,149]. A substance P receptor antagonist (SPRA) that reduced intra-abdominal adhesion formation in rats also decreased peritoneal MMP activity [150]. These preclinical studies have provided some insight into the potential impact NK-1R mediators can have in mitigating post-surgical adhesion formation, but more robust data is needed to further advance them into the clinical setting.

### 3.5. Lubricin

Lubricin is a mucin-like proteoglycan that has been found in various tissue compartments, including synovial fluid [151] and the eyes [152]. Many in vitro and in vivo studies have found lubricin to have anti-adhesive and anti-inflammatory properties [153]. A recent study by our group showed for the first time that lubricin exists in human pericardial fluid [43]. We found that lubricin localizes to the pericardial mesothelial layer and prevents myofibroblast attachment and activity. Using a porcine model, our group also showed that the removal of pericardial fluid is associated with severe pericardial adhesions, while adding lubricin to the pericardial space resulted in fewer and less severe adhesions in the early post-operative stages [43]. Further study and validation are required to delineate the mechanism of action of lubricin in preventing adhesion formation and its translation into clinical practice.

### 3.6. Chymase Inhibitor and Sodium Cromoglycate

Chymase is an inflammatory mediator released from mast cells and is involved in tissue fibrosis [154,155]. The administration of various chymase inhibitors has resulted in lower adhesion formation and decreased TGF-β levels in multiple preclinical animal models of peritoneal and pericardial adhesions [156,157,158,159,160,161,162]. However, in a hamster model of peritoneal adhesions, moderate to severe adhesions persisted in 18% of hamsters regardless of chymase inhibitor use, suggesting that the dosing and administration of the inhibitor requires further optimization [156]. On the other hand, sodium cromoglycate stabilizes mast cell membranes and prevents the release of histamine and other biochemical mediators. Intra-peritoneal instillation of sodium cromoglycate in a rabbit model of pelvic adhesions demonstrated efficacy in reducing the incidence of adhesion formation, both individually and when combined with saline, dexamethasone, and aprotinin [163]. As is the case with many pharmaceutical agents discussed here, more robust preclinical and clinical studies are needed to better understand the potential impact these agents can have in preventing or minimizing post-operative adhesion formation.

### 3.7. NSAIDs and Anti-Inflammatory Drugs

Administration of non-steroidal anti-inflammatory drugs (NSAIDs) has been shown to decrease peritoneal, pelvic, and tendon adhesions in rabbit, hamster, guinea pig, and porcine models [164,165,166,167,168,169,170,171,172,173,174,175,176]. Although the exact mechanism by which they exert this effect is unclear, it is likely secondary to their inhibition of cyclooxygenase 2 (COX-2), which leads to decreased production of prostaglandins in the inflammatory cascade [177]. In murine models of peritoneal adhesions administration of COX-2 inhibitors, such as celecoxib, tenoxicam, and pentoxifylline, reduced post-operative adhesions through their anti-angiogenic, anti-inflammatory, and antioxidant effects [178,179,180,181,182,183]. Furthermore, NSAIDs block the production of thromboxanes, which are reportedly involved in biochemical pathways leading to adhesion formation [184]. Selective inhibition of thromboxane production via NSAID administration can be a potential strategy for reducing adhesion formation and severity [169]. Nevertheless, given their systemic side effects such as peptic ulcer disease, acute renal failure, electrolyte and fluid abnormalities, increase risk of adverse cardiovascular events, hepatotoxicity, bronchospasm, cognitive dysfunction, and various skin reactions [185,186,187,188,189,190,191], a scenario where NSAIDs can be delivered locally and show the same anti-adhesive efficacy would be optimal. Indeed, early studies of locally injected NSAIDs and anti-thromboxane agents in a rabbit uterine horn model were efficacious in reducing the severity of adhesion [184]. However, no in-human trials have been performed to examine the efficacy of NSAIDs in the reduction of post-surgical adhesions. Now, more contemporary studies have moved away from local or systemic NSAID administration and instead imbued NSAIDs into inert physical barriers. This approach for adhesion prevention will be discussed below.

### 3.8. Alcohol

Ethanol has been assessed in a porcine pericardial adhesion model [192,193]. One study supplemented the diets of the subjects with alcohol and compared the effects with a sucrose-supplemented diet group. There was evidence of fewer adhesions, thinner pericardial thickness, and decreased intra-myocardial fibrosis in the alcohol group [192]. Groups have yet to explain the rationale behind these observations, and there are currently no studies that have considered the anti-adhesive properties of alcohol in a clinical setting.

### 3.9. Small Molecule Inhibitors

Small molecule inhibitors are gaining more attention in various clinical settings. Pirfenidone, a novel small molecule inhibitor, acts by reducing inflammatory cytokines such as TNF-α, monocyte chemotactic factor-1, IL-1β and IL-6; down-regulating profibrotic growth factors, including TGF-β; and reducing lipid peroxidation and oxidative stress [194]. In a postoperative peritoneal adhesion rat model, pirfenidone has demonstrated efficacy in preventing adhesion formation [195]. In a murine model, actin modulators, including CK-666, Rhosin, and Golgicide A, and a calcium channel antagonist, Bepridil, have been found to robustly inhibit adhesion formation following daily peritoneal injections for five days [196]. Moreover, a single early localized administration of the above compounds in a 2% cellulose compound was also successful in preventing adhesion formation [196]. As a proof of concept, this study suggests that calcium signalling can potentially be targeted in a precise manner in order to prevent adhesion formation.

Trametinib is a small molecule kinase inhibitor of mitogen-activated extracellular signal regulated kinase (MEK) 1 and 2. In a mouse model of abdominal adhesions, trametinib effectively blocked macrophage to myofibroblast transition in vitro and markedly diminished adhesion formation in vivo, likely by inhibiting the activation of Erk1/2 [197]. This data suggests that activation of Erk1/2 signalling pathways can be a common pathophysiological mechanism in adhesion formation and other fibrotic reactions. The activation of these signalling pathways induces the production of collagen-containing extracellular matrix (ECM) as well as a transition to a myofibroblast phenotype in resident cells [198]. The small molecular inhibitor QLT-0267 has been reported to attenuate fibrin-induced IL-1β, IL-6, TNFα, and VEGF-A expression in polymorphonuclear cells [199]. This study demonstrates that fibrin, the key component of the initial adhesion matrix, not only apposes two peritoneal surfaces but could also augment peritoneal inflammation and cause peritoneal adhesions. The study also indicates that fibrin-induced cytokine production can be involved in the mechanism of peritoneal adhesion formation.

Work has demonstrated the efficacy of T-5224, a selective small molecule activator protein (AP)-1 inhibitor used in a variety of fibrotic pathologies and shown to block early and late AP-1-induced cytokine responses [200]. The gene *JUN* has recently been identified as a transcriptional master regular of fibroblasts and has been shown to be expressed in human adhesion fibroblasts [40]. *JUN* expression is an early promoter of abdominal adhesions, upregulating fibrotic signalling pathways in both mouse and human adhesion formation models [40]. T-5224 has also been shown to suppress *JUN* signalling in mouse adhesion fibroblasts in vitro, and intra-abdominal application in vivo demonstrated a dramatic decrease in adhesion formation, with histological examination revealing thinner and less fibrotic adhesions.

### 3.10. Hormones and Other Pharmacological Agents

Estrogen is a steroidal hormone mainly produced by ovaries, placenta, and to a lesser extent the adrenal cortex. Estradiol has been reported to have a significant effect on the formation of pelvic adhesions after myometrial operation in non-human primates [201]. Intraperitoneal administration of estrogen has been shown to reduce abdominal adhesion formation in a rat model [202]. A systemic review examining the efficacy of estrogen therapy in patients with intrauterine adhesions concluded that estrogen therapy is beneficial to patients with adhesions regardless of the severity [203].

Ghrelin is a gastric peptide hormone that has been identified to have anti-fibrotic and anti-inflammatory actions [204]. Intraperitoneal administration of exogenous acylated ghrelin has been found to reduce the formation of post-operative abdominal adhesions in a murine model [205]. This anti-adhesion effect has been confirmed and mechanistically identified to involve the down-regulation of the pro-inflammatory gene and protein expression, including TGF-β3 and TGFβ-R2 [206]. Other methods, including anticoagulants, antioxidants, antihistamines, melatonin, or neutralizing of fibrinolytic inhibitors, and inflammatory cytokines have also been investigated [207,208,209,210,211,212,213,214,215]. Although some of these agents showed positive results in animal studies, no conclusive data supporting their efficacy have been reported from the aforementioned studies.

While the use of pharmaceutical agents alone has potential to prevent tissue adhesion formation, there are certain drawbacks related to off target and adverse effects. For example, fibrinolytic agents can lead to dysregulation of the coagulation system and uncontrolled bleeding [216], whereas NSAIDs contribute to immune-suppression and a delayed healing process as well as a host of potential adverse effects [217,218]. While agents targeting various mediators in the RAAS are typically well tolerated, RAAS activity is regulated systemically and locally in a manner in a variety of systems and tissues [219]. The potential for the development of adverse and unanticipated effects is high due to the intertwined nature. Regarding HIF inhibitors, the use of many is dampened by their off-target effects, affecting different pathways in DNA replication, cell division, or cell signaling [220]. HMG-CoA reductase inhibitors have been hypothesized to influence intracellular signaling pathways and immune processes [221,222]; their several pleiotropic off-target effects have been investigated in cancer therapy by delaying DNA repair and inducing senescence [223]. Chymase inhibitors have off-target effects linked to various biological pathways such as the complement system, intrinsic, and extrinsic pathways of coagulation cascade, and fibrinolytic system [224]. These exemplify significant issues that have contributed to the limited clinical translation of pharmaceutical agents that have been used for preventing or minimizing post-surgical adhesion formation.

## 4. Inert Polymers in Adhesion Prevention Strategies

Given the aforementioned challenges with potential anti-adhesive drugs, including lack of clinical efficacy, groups have assessed the safety, feasibility, and efficacy of physical barriers that can be used to prevent or lessen the severity of post-operative adhesions.

### 4.1. Barriers

Barriers are based on the hypothesis that preventing physical contact between two injured tissue or cell surfaces will prevent adhesion formation. These products can be generally classified into solid barriers, gels, and solutions. Bioactive functional barriers have been developed by embedding anti-adhesive drugs into barriers. Research has shown that key bioactive mediators driving post-surgical adhesions are present up to 7 days after an operation. An ideal barrier would be placed on the site of injury and be degraded safely after 7 days to prevent future complications. However, the introduction of barriers into clinical practice has been restricted by limitations in preparation and application, insufficient pliability, complex product fixation techniques, the need for absolute hemostasis, and incompatibility with laparoscopic surgical procedures [225]. Table 2 summarizes barrier types and reasons why they have shown some efficacy as tissue adhesion barriers.

#### 4.1.1. Natural Polymers

##### Hyaluronic Acid (HA) Based

Barriers containing hyaluronic acid (HA), also referred to as hyaluronan or hyaluronate, are a commonly seen type of adhesion prevention. HA is a naturally occurring glycosaminoglycan and forms a highly viscous solution to coat serosal surfaces. The mechanism by which HA solutions prevent adhesion formation is unknown, but may be due to a cytoprotective effect on mesothelial surfaces [226]. HA solutions have been shown to reduce adhesions following abdominal, orthopedic, and cardiac surgery in animal models [226,227,228,229,230]. Topical administration of HA has been reported to be efficacious in the prevention of post-operative pericardial adhesions in canine and monkey models, and appears to be safe in doses five times the amount required for adhesion prevention [226]. One study showed that applying 0.1% sodium HA in a canine model caused no clinically significant pericardial adhesions compared to 1% carboxymethyl cellulose, which had 20% adhesions [231]. In another study, HA was compared to paraffin in preventing intra-peritoneal adhesions in rats and found no significant difference between the two, although the presence of both significantly reduced adhesion formation [232].

As a natural polymer, HA experiences rapid clearance from the applied site due to high blood and body fluid solubility. However, this potential benefit may also lead to reduced efficacy in minimizing post-surgical adhesion formation and is considered a critical hurdle. Cross-linking polymers with chemicals, heat, or light can be a simple strategy to increase their stability in the body. However, loss of specific functional groups in tissue interaction minimizes tissue adhesiveness after cross-linking procedure [233]. Cross-linked HA hydrogel is reported to reduce post-operative adhesion prevention in a mouse uterine horn model [234], whereas a recent human study found the gel to reduce intra-uterine adhesion formation [235]. It has been suggested that semi-interpenetrating polymer networks of certain natural polymer combinations can be a solution that compensates for the shared problem of natural polymers [236].

##### Cellulose Based Barriers

Carboxymethyl cellulose (CMC) is a cellulose derivative that forms the basis of two commonly used and commercially available adhesion prevention barriers: Interceed (Johnson & Johnson, Cincinnati, OH, USA) and Seprafilm (Genzyme, Cambridge, MA, USA). Interceed is composed of oxidized regenerated cellulose (ORC), which degrades within 2 weeks after placement [36]. Barriers using ORC are commonly believed to offer an inert and inactive barrier to cellular adhesions, as ORC does not appear to alter the signalling behaviour of mesothelial cells directly [237]. However, Interceed has also been shown to increase expression of tPA in mesothelial cells and increase the tPA:PAI-1 ratio, signifying an overall increase in fibrinolytic activity [238], as well as altering the macrophage immunologic response [239].

Interceed has been studied in animal models in combination with heparin and has been shown to significantly reduce adhesion formation in rabbit uterine horn [240,241] and pericardium [242]. Similarly, other human trials generally find that Interceed reduces the severity, extent, and/or incidence of pelvic adhesions [243,244,245,246,247,248]. A review of 15 controlled trials in humans reported that Interceed has superior performance compared to Seprafilm in reducing the incidence of adhesion formation [249]. In contrast to these promising results, several murine preclinical models have found no effective prevention of abdominal adhesions [250,251,252,253]. A meta-analysis of 7 human studies of nearly 400 patients undergoing laparoscopic pelvic surgery found that the barrier significantly reduced extent and severity of adhesions, but not adhesion incidence [254].

Interceed has several limitations, including that all blood and peritoneal fluid [255] in the abdomen must be cleared before application, while folding or misplacement of Interceed can also induce adhesion formation [256]. Finally, although large clinical studies generally conclude that Interceed is biocompatible and not associated with an increase in adverse events [249,254], the product has been reported to induce a large leukocyte response and stimulate mesothelial cell sloughing in mice [257].

Seprafilm is a commercially-available translucent and temporary adhesion barrier for preventing adhesions. Seprafilm consists of a solid sheet of biodegradable chemically-modified sodium hyaluronate and carboxymethyl cellulose that physically separates tissue surfaces. It is one of the most studied and implemented anti-adhesion products [258] and has shown efficacy in reducing abdominal adhesions in various animal models [259,260,261,262,263,264,265,266,267,268,269,270,271]. Furthermore, Seprafilm has been explored in preclinical studies of pericardial [229,272,273,274] and pleural adhesions [258]. In a mouse model of redo laparotomy, Seprafilm was found to be effective in preventing adhesions when applied during redo laparotomy and the anti-adhesive effects were most pronounced when applied onto dense adhesions at the time of the redo surgery [275].

Randomized, controlled, human trials with greater than 5000 total patients enrolled show that Seprafilm has some efficacy in reducing the incidence, severity, extent, and/or area of abdominal adhesions [243,255,276,277,278,279,280,281,282,283,284]. However, other human studies suggest that Seprafilm only decreases adhesion severity, not incidence [285,286,287]. A review of 15 randomized controlled trials examining the prevention of pelvic adhesions in female patients reported that Seprafilm had no efficacy in adhesion prevention versus controls [249]. Similarly, studies have failed to consistently confirm the safety of Seprafilm. A meta-analysis of the efficacy and safety of Seprafilm in general surgery patients revealed that abdominal adhesions decreased significantly after use, but it was found to have no effect on postoperative intestinal obstruction and was found to increase abdominal abscesses and anastomotic leaks [288]. Multiple reports also note that Seprafilm is brittle, and suffers from easy adherence to any moist surface, notably surgeons’ gloves during operation [289,290]. Further, the product cannot easily be affixed to irregularly shaped tissue planes after surgery [275]. Despite these limitations, Seprafilm remains the standard of care in adhesion studies, although few would list it as a definitive solution for the prevention of adhesions [291].

##### Chitosan Based

Chitosan is a linear polysaccharide and a common substance found in barrier products. Chitosan-containing barriers are available as a film, gel, or combined with cellulose and seaweed polysaccharides. Several studies demonstrate that chitosan-based products decreased coverage and severity of adhesions in rabbit, porcine, and murine models in cardiac, abdominal, and gynecological surgeries [292,293,294,295,296,297,298,299]. To our knowledge, chitosan itself is yet to be employed in clinical use in humans. Chitosan-based polymers exhibit excellent hemostatic ability, used in commercially available hemostatic products [300]; this characteristic has been leveraged in adhesion prevention. The anti-adhesive mechanism is unclear, but it is hypothesized that it inhibits fibroblast activation and interrupts fibrin matrix formation. Chitosan is most commonly used in combination with other polymers, producing a product known as N,O-carboxymethyl chitosan.

N,O-carboxymethyl chitosan has shown efficacy in the reduction of adhesions in the peritoneum and pericardium of rats and rabbits [299,301,302] as well as in patients undergoing pelvic laparoscopy [303]. A similar product, oxidized dextran/N-carboxyethyl chitosan (Odex/CEC) showed significantly decreased adhesion formation in comparison to control in a post-operative abdominal rat model [304]. Novel preparations have used silkworm pupa to generate N,O-Carboxymethyl chitosan, which has shown efficacy in a rat post-operative peritoneal adhesion model and demonstrated lower levels of TGF-β1 expression [305]. A combination polymer barrier, utilizing transglutamine to crosslink carboxymethyl chitosan, carboxymethyl cellulose, and collagen has been found to reduce adhesions in an intra-peritoneal adhesion rat model [306]. This study demonstrated the enhanced mechanical properties and improved biodegradability of the combination polymer, suggesting that biodegradable physical barriers can be more efficacious when incorporated with polymers. Similarly, a combination of N,O-carboxymethyl chitosan/oxidized regenerated cellulose (N,O-CS/ORC) composite gauze has been shown to significantly prevent post-surgical peritoneal adhesions in rats [307], while also serving as an excellent hemostatic agent [306]. This provides an advantage over commercially-available barriers such as Interceed, which is applied only when the entire area is completely hemostatic, an issue that is not trivial in the surgical field.

##### Natural Films

REPEL-CV (SyntheMed, Iselin, NJ, USA) is another commercially-available film-based anti-adhesive product that consists of polyethylene glycol and polylactic acid. In cardiothoracic surgeries, REPEL-CV has shown promising results in reducing the formation and severity of post-operative adhesions. These findings have been shown in animal models as well as in human trials of pediatric patients requiring staged sternotomies [308,309,310,311,312,313,314]. REPEL-CV received FDA approval for use in pediatric and young adult patients likely to need repeat heart surgery. Similar to other barriers, the product provides mechanical separation between opposing tissue surfaces. It has also been suggested that the film acts as a scaffold for tissue regeneration, which ultimately eliminates adhesions [315]. SurgiWrap (MAST Biosurgery USA, Inc. San Diego, CA, USA) is another commercially-available polylatic acid film, which has been associated with significantly reduced incidence and severity of post-operative intra-abdominal adhesions in rat [316] and porcine models of abdominal adhesions [317]. In a human trial, SurgiWrap was associated with reduced peristomal adhesion severity in patients undergoing laparoscopic colorectal cancer surgery [318].

#### 4.1.2. Synthetic Polymer Meshes

Non-degradable synthetic polymers have also served as primary forms of barriers against adhesion formation. These include silicone, polyethylene (PE), and expanded polytetrafluorethylene (ePTFE) [10]. Non-degradable polymers require a secondary surgery to remove them, which in itself poses risks of adhesion formation or greater surgical complications due to repeated surgery. Further issues with these mesh polymer barriers include suturing, which was required to affix the barrier due to low adhesiveness with surrounding tissue, and difficulty in application to complex surfaces and in laparoscopic applications due to high flexural rigidity [10].

Gore-Tex (W. L. Gore & Associates, Newark, NJ, USA) is a commercially-available and commonly used synthetic surgical membrane, composed of non-resorbable expanded polytetrafluorethylene (ePTFE). This inert and permanent barrier acts by preventing cellular growth to prevent adhesion formation [282], and has been used predominantly in pelvic, peritoneal, thoracic, and pericardial surgical applications [256,319,320,321,322]. In contrast to other solid barrier products, Gore-Tex must be sutured in place, which can increase adhesion incidence directly and indirectly by prolonging operation time [323]. A 1993 study suggested that Gore-Tex decreased adhesions around the internal mammary artery in a goat model [324]. Further studies using ePTFE have shown some efficacy in adhesion prevention in murine and rabbit models [253,323,325] and a randomized clinical trial examining pelvic adhesions post-myomectomy [326].

Gore-Tex has shown anti-adhesive performance superior to Interceed in mouse and monkey models of pelvic adhesions as well as human studies [249,257,327,328]. There is ongoing debate regarding the lack of removal or absorption of ePFTE, which contributes to long-term safety concerns about the mesh, particularly noting the concerns of an extensive inflammatory reaction resulting in fibrous capsule formation [329]. Studies that do not remove the ePFTE barrier in vascular and pericardial grafts found no significant long-term adverse effects [330], but leaving a foreign body in the surgical field can predispose patients to infections over time [331]. A prospective multi-centre observational study investigating the long-term use of ePTFE without removal reported only one case of post-operative infection, which did not necessitate removal of the membrane [332].

In comparison to polypropylene and polyester, one study found that ePTFE elicits a negligible inflammatory response [55]. However, another study revealed that, due to its non-absorbable nature, ePFTE causes higher rates of inflammation and macroscopic pericardial adhesions compared to a collagen-sponge patch [333]. A study examining polypropylene mesh coated with the commercial product CoSeal (Baxter Healthcare Corp., Deerfield, IL, USA), a re-absorbable hydrogel of polyethylene glycol, has shown superior reduction of adhesion formation in rabbits compared to uncoated meshes [334]. Generally, the literature demonstrates that bioprosthetic meshes have fewer lower-grade adhesions than synthetic mesh [55]. These studies provide evidence that bio-absorbable, natural polymers such as polyvinyl alcohol, polylactic acid, and polyethylene glycol-based biomaterials, are preferable in decreasing adhesion formation. Further studies have been conducted to optimize mesh barriers. One innovative approach that can compensate for the conventional limitations of sheet, mesh, and film type barriers involves the fabrication of barriers with well-organized, porous surfaces that have the capability to absorb blood and body fluids in micropores, thus inducing capillary force [335,336]. Another approach for addressing drawbacks is the synthesis of copolymers. This strategy involves sourcing monomers with a low glass transition temperature, permitting flexibility of the sheet when at body temperature. These copolymers have demonstrated convenience and positive results in adhesion prevention [337].

Collagen-coating on applied meshes has demonstrated enhanced biocompatibility [338], where intra-peritoneal adhesion formation was reduced at day 7 following the use of a collagen-coated mesh [339]. Meshes and products using collagen have been reported to be highly effective in reducing post-operative abdominal adhesions in animal and human studies [340,341,342,343] achieving better clinical efficacy than Interceed [344]. These studies have formed the basis for commercial collagen-based or coated mesh barrier products, which include Alloderm (LifeCell, Branchburg, NJ, USA) [345], Parietex (Covidien, Dublin, Ireland) [346,347,348], Permacol (Covidien, Dublin, Ireland) [349,350,351], and Surgisis (Cook Group, Bloomington, IN, USA) [351,352,353,354].

#### 4.1.3. Collagen Sheets

Collagen sheets are a relatively new form of anti-adhesive barriers. They are bio-absorbable membranes that also act as a scaffold for new cells. In abdominal adhesion models in rats [344] and a rabbit uterine horn abrasion model [355], collagen membranes have been more efficacious than Interceed. Collagen sheets applied to reduce post-operative pericardial adhesions in animal models also resembled native pericardial membranes at 24 weeks post-operation, suggesting a seamless integration [331,356]. A pilot study utilizing a porcine model of pericardial adhesions found fewer macroscopic and microscopic adhesions in all regions after collagen sheet application, although these differences were not statistically significant [357]. COVA (Biom’Up, Lyon, France) is a collagen membrane that has shown feasibility in the prevention of severe pericardial adhesions in pediatric patients undergoing cardiac surgery [358] and in sheep [359]. A recent prospective multicenter study in patients undergoing two-stage abdominal surgeries found that COVA is safe and showed efficacy in reducing post-operative abdominal adhesions [360]. A HA-collagen membrane has also demonstrated efficacy in a rat model of peritoneal adhesions [361]. TachoSil (Nycomed Austria GmbH, Linz, Austria), a hemostatic collagen sponge, has also been found to be superior to Gore-Tex in a rabbit pericardial adhesion model due to its ability to decrease adhesion formation and its scaffolding properties [331,333].

#### 4.1.4. Composite Polymers

Composite meshes have shown to decrease visceral adhesions in the chest wall [362] and post-operative adhesions in a rat model [363]. The commercially-available tri-component resorbable mesh, Prevadh (Sofradim Production, Trevoux, France) is composed of polylactic acid, lyophilized porcine collagen, and a hydrophilic collagen film. The product has been effective in preventing intraperitoneal [364] and pleural [365] post-surgical adhesions in rat models. In human trials, Prevadh significantly reduced pelvic adhesion incidence and severity compared to Ringers lactate solution in patients undergoing gynecologic surgery [366]. A prospective multi-center study of patients undergoing both open and laparoscopic general surgery has reported the safety of Prevadh for the prevention of intraperitoneal adhesion formation [367], although a large randomized control trial is yet to be performed.

Composite polymer solutions have some drawbacks, including rapid degradation and unstable biofixation, hence reducing their efficacy [368,369]. Another composite copolymer, d,l-polylactide-ε-caprolactonetrimethylenecarbonate, has been shown to decrease adhesions in a post-operative peritoneal rat model, and is suitable for laparoscopic surgery [370], although further validation and investigation is warranted. Current studies have produced more advanced polymeric hydrogels. Stapleton et al. have shown that a dynamically crosslinked supramolecular polymer–nanoparticle hydrogel is superior to Interceed and Seprafilm in reducing the severity of pericardial adhesions in a rat model [371].

#### 4.1.5. Spray Type Barriers

In efforts to have a barrier that is user friendly, spray-type barrier systems have been developed. In infants undergoing cardiac surgery, a sprayable polymeric matrix has been reported to reduce the incidence and severity of pericardial adhesions [372]. CoSeal Surgical Sealant (Baxter Healthcare, Deerfield, IL, USA), a sprayable synthetic polymeric hydrogel consisting of polyethylene glycol, has been reported to reduce pericardial adhesion severity in rabbits [373] and pediatric patients undergoing repeat heart surgery [372]. A European multicenter study employing the same product reports similar results, however, six adverse events (cardiac fibrillation, pericardial effusion, mediastinitis, supervisor vena cava occlusion, and two cases of cardiac tamponade) were attributed to the product and the study lacks controls [374].

SprayShield (Covidien PLC, Dublin, Ireland) is another polyethylene glycol-based system that has demonstrated anti-adhesive properties in a preclinical porcine model [375] and in a clinical trial of patients undergoing myomectomy [376]. However, another human study found no difference in post-surgical adhesion formation between control and treatment groups [377], although the study has a small sample size and significant confounding factors. A sprayable version of SepraFilm, known as SepraSpray (Genzyme, Cambridge, MA, USA), consists of a powdered form of sodium HA and carboxymethylcellulose. SepraSpray has demonstrated safety and efficacy in preventing abdominal adhesions in rats [378] and in randomized human trials [379,380].

A recently developed spray-type barrier, TCD-11091, was assessed in a randomized, controlled trial of 126 patients undergoing laparotomy, where it was found to reduce the incidence of adhesions [381]. Actamax (Actamax Surgical Materials LLC, Wilmington, DE, USA) is another new sprayable adhesion barrier, which is formed by mixing aqueous dextran aldehyde and polyethylene glycol amine polymers to form a temporary hydrogel. The hydrogel is designed to prevent apposing tissue surfaces from contacting during the immediate post-operative peritoneal healing period (3–5 days) when adhesions are most likely to form [382]. Data from the first in-human randomized control trial suggests the product is safe and effective in reducing post-operative adhesion development, particularly following myomectomy [383]. Although the spray delivery systems facilitate easier use and can incorporate various products, none have been consistently effective in preventing post-operative adhesion formation [384,385,386,387].

#### 4.1.6. Solutions as Barriers

Irrigating the surgical field using solutions has been believed to aid in preventing or reducing post-surgical adhesion formation. These solutions are typically composed of crystalloids or high-molecular-weight dextran. The use of irrigating solutions is known as hydroflotation, whereby contact between surfaces is reduced to minimize adhesion formation [321]. A meta-analysis of 259 articles examining post-operative adhesion incidence after pelvic surgery concluded that crystalloid irrigation does not reduce adhesion formation [388]. The use of dextran has also been met with conflicting results [389,390]. The high viscosity and long half-life of dextran in the peritoneal cavity also raises concerns regarding potential hemodynamic compromise due to excessive fluid shifts [321].

Adept Adhesion Reduction Solution (Innovata plc, Surrey, UK) is a commercially-available 4% icodextrin solution made of an α(1–4)-linked glucose polymer that acts via hydroflotation. Its intra-peritoneal administration acts as a colloidal osmotic agent, which retains a fluid reservoir within the peritoneal cavity and creates physical separation between peritoneal surfaces. Minimizing surface apposition during the cycle of fibrin deposition and matrix formation has been suggested to reduce adhesion formation [391]. In preclinical animal models, Adept has been promising in reducing the severity and extent of post-surgical adhesion formation [392,393,394]. Although Adept has been found to be safe [395], similar to other hydroflotation solutions, it has had limited efficacy in randomized clinical trials [391,396,397].

#### 4.1.7. Xenograft Membranes

Xenograft membranes include equine pericardium, bovine pericardium, and porcine small intestine submucosa. Little has been published regarding their anti-adhesive properties, although these materials have shown early promising results [398,399]. A combined application of acellular bovine pericardium and hyaluronic acid in a rabbit model has shown efficacy in the reduction of postoperative pericardial adhesions [400].

## 5. Functional Biomaterials for Adhesion Prevention

Barrier approaches that employ the use of inert biomaterials have been shown to be effective in animal and human studies. Promisingly, there is a high potential to transform an inert barrier or solution into an effective drug-loaded carrier. Biocompatible polymers have evolved into bioactive and bioinert materials and have been utilized in a variety of biomedical applications such as complex tissue engineering, cell, drug, and gene delivery systems, and organoid formation. A better understanding of the dysregulated tissue healing process that leads to adhesions, current biomaterials can be engineered to be more efficacious in adhesion prevention. Emerging strategies include the incorporation of anti-inflammatory, anti-coagulative, and fibrinolytic agents, as well as growth factors into different types of barriers [7]. Table 3 summarizes the agents and pharmaceuticals that have been used in preclinical and clinical studies along with their indication and findings.

### 5.1. Integrated Pharmaceuticals in Barriers

The use of bioactive barriers offers a unique and compelling approach to preventing post-surgical adhesion formation. Various bio-modifications can be used to transform barriers from inert and inactive walls into active signalling materials that can be instructive to cells, the local environment, and serve as highly localized and effective platforms for drug delivery. However, a detailed analysis of the optimal delivery methods and the choice of which agents to use will be necessary to develop effective integrated pharmaceutical-barrier products [343].

A thermo-sensitive hydrogel augmented with tPA combined barrier functions with sustained anti-adhesive drug release. In a rat repeated-injury peritoneal adhesion model, the administered tPA-hydrogel successfully promoted mesothelial regeneration on the abdominal wall and decreased PAI-1 levels in peritoneal lavage fluids, leading to decreased fibrin formation [401]. These results indicate the efficacy of targeting the fibrinolytic system in adhesion prevention and the potential to enhance drug delivery via the use of a thermosensitive hydrogel. Lidocaine has recently been shown to have an anti-adhesive effect when loaded onto a poloxamer-alginate-CaCl2 (PACM) barrier in a rat planar incision model [402]. Advanced fabrication methods have allowed for the preparation of a lidocaine-enriched alginate/CMC/polyethylene oxide (PEO) nanofiber film by electrospinning, which facilitated drug release by cross-linking [403]. Although only tested in vitro, this approach demonstrates a promising strategy for the development of sustained drug delivery methods in preventing adhesions.

A thermo-sensitive hydrogel barrier, known as C2.5T1M0.2 thermo-gel, has been developed by combining mitomycin C (MMC) with modified tempo-oxidized nano-cellulose and methyl cellulose. MMC is a DNA alkylating antitumor antibiotic, which inhibits in vitro fibroblast proliferation with anti-fibrinolytic activity [404]. In a rat model of cecal abrasion, de novo adhesions were effectively prevented and reperitonealization was also observed [405]. The efficacy of this hydrogel can likely be attributed to the combination of a physical barrier with MMC, which is reported to upregulate TNF-α, and downregulate collagen and fibronectin gene expression, although the specific cellular effects require elucidation [405]. Similar prevention of pericardial adhesions was seen in a rat model of post-operative adhesions [406]. Another study has also reported the inhibition of peritoneal adhesion formation in a rat model utilizing a novel HA and CMC-sodium cross-linked hydrogel loaded with oxaliplatin, a platinum-containing chemotherapy drug [407,408]. The effect of oxaliplatin on adhesion formation has not been fully evaluated, and more studies are warranted to delineate the anti-adhesive mechanisms of this hydrogel. Moreover, the co-administration of a HA/CMC barrier material with an NK-1R antagonist in a rat model has demonstrated increased anti-adhesive efficacy at the application site and significantly reduced adhesions at remote sites [409]. Although more detailed investigations are needed, this combination represents an emerging and promising concept in more effectively preventing post-operative adhesion formation throughout the peritoneum.

Ibuprofen, an NSAID, has also been used to augment copolymer films in order to reduce adhesion formation by preventing mass migration of inflammatory cells in a rat peritoneal adhesion model [410]. Ibuprofen has been loaded into electrospun poly(L-lactic acid)-polyethylene glycol fibrous membranes, which prevented tissue adhesion and inflammation in a chicken model of tendon adhesions [411]. Electrospun poly(L-lactide) (PLLA) and its copolymers have been prepared in combination with various pharmaceuticals, such as the antibacterial beta-lactam cefoxitin sodium [412], the NSAID celecoxib [413], combinations of hemostatic, antibacterial, and antiadhesive agents [414], and others [415,416]. Similarly, naproxen has been added to a chitosan hydrogel and shown to prevent post-surgical peritoneal adhesions in a rat model [417]. While the anti-adhesive mechanisms of NSAIDs continue to be investigated, this study demonstrates the feasibility of using barrier products to serve as both a drug-delivery platform and as an anti-adhesive therapeutic. However, it is difficult to achieve long-term drug release using current electrospun fibrous membranes of biodegradable polymers because of their large surface area to volume ratio.

### 5.2. Nanoparticles and Gene Therapy

Nanoparticles have been suggested as controlled drug delivery carriers because of their unique physical properties, which include uniform and modifiable particle and pore size; high surface area and large pore volume; and superior biocompatibility [418]. Silver nanoparticles (AgNPs) have been loaded into electrospun fibrous membranes composed of PLLA, where an in vitro study demonstrated that this novel membrane suppressed adhesions and the proliferation of fibroblasts in addition to having antibacterial effects [419]. Ibuprofen has also been integrated into AgNP-PLLA electrospun membrane, which showed enhanced adhesion prevention and anti-proliferation effects in a chicken tendon adhesion model [420]. In a rat model of tendon adhesions, a novel preparation of pre-formulated dextran glassy nanoparticles (DGNs) were loaded with basic fibroblast growth factor (bFGF or FGF-2) [421]. The encapsulation of bFGF into these fine, polysaccharide glassy particles was done to preserve the stability and biological activity of the growth factor. The incorporation of bFGF-DGNs into PLLA fibers can serve to control the release of bFGF from this barrier method while leveraging the physical separation capabilities of the polymer. This study highlights the potential to leverage bioactive mediators that have been shown to be dysregulated in post-surgical adhesion formation, such as bFGF, while utilizing the benefits of inert biomaterial barriers. Sustaining the biological activity of bFGF through this method was shown to enhance tendon healing and simultaneously prevent peritendinous adhesion in vivo [421].

Polylactic-co-glycolic acid (PGLA) nanoparticles have also been used to modulate gene expression via sustained gene delivery of TGF-β1 mi-RNA plasmids in a chicken tendon adhesion model [422]. By targeting and downregulating TGF-β1, this study shows that using a plasmid-nanoparticle method as a non-viral vector for gene therapy can potentially be beneficial in adhesion prevention. Similar studies have demonstrated less severe adhesion grading in the same chicken tendon model upon treatment with PGLA-TGF-β1 mi-RNA RNAi plasmids, although the ultimate strength of tendons repaired with plasmids was significantly lower [423]. Nanoparticles have also been utilized to deliver bFGF and VEGF genes, demonstrating up-regulated protein expression in transfected tenocytes and significantly enhanced healing ability [424]. The use of biologically-active substances loaded into nanoparticle membranes offers a great opportunity to deliver anti-adhesive therapeutics in a targeted and personalized manner, hence addressing the systematic side effect issues of many other agents.

**Table 3:** Materials demonstrating efficacy in human or animal models as per clinical indication of anti-adhesion results.

Type	Material	Company	Model	Reference
**Natural Polymers** **(biodegradable)**				
	**Alginate**		Rat (peritoneal)	[233,425]
	HA-mildly crosslinked alginate hydrogel		Rat (peritoneal)	[426]
	Pluronic mixtures/crosslinked alginate	Guardix-SG (Genewel)	Rat, Rabbit (peritoneal)	[427,428]
			Rabbit (pericardial)	[429]
			Rat (pelvic)	[430]
			Human (abdominal)	[431]
	HA-crosslinked alginate (powder)			[432]
	**Gelatin**		Rat (peritoneal)	[433,434]
			Canine (abdominal)	[435]
			Canine (pelvic)	[436]
	Chitostan-gelatin films		Rat (peritoneal)	[437]
	Gelatin-polyglycolic acid sheets		Canine (pericardial)	[315]
	Genipin-crosslinked gelatin microspheres		Mouse (peritoneal)	[438]
	**Gellan Gum**		Rat (peritoneal)	[439]
	**Poly-γ-glutamic acid**		Rat (peritoneal)	[440]
	**Fibrinogen/thrombin** (Fibrin glue)	Evicel (Johnson & Johnson Medical)	Rat, Rabbit, Human (pelvic)	[441,442,443,444]
	Fibrin glue		Canine (pericardial)	[445]
**Hyaluronic Acid (HA) Based**				
	Auto-crosslinked HA		Rat (peritoneal)	[446,447,448]
			Human (pelvic)	[236,449]
	Crosslinkable HA derivative-alginate		Rat (peritoneal)	[450]
	Phenolic hydroxyl modified HA hydrogel		Mouse (abdominal)	[451]
	HA-adipicdihydrazide-HA-aldehyde		Rabbit (peritoneal)	[452]
	HA-hydrazide-celluloses-aldehyde [Celluloses: HA, CMC, hydroxypropylmethylcellulose (HPMC), methylcellulose (MC)]		Mouse (peritoneal)	[453]
	**Pullan**		Rat (peritoneal)	[454]
**Synthetic Polymers**				
	**Polyethylene glycol (PEG)**		Rat (peritoneal)	[455]
	PEG-aliphatic polyester		Rat, Rabbit (abdominal)	[456,457]
	PEG dicarboxylate-poly(ethylene oxide) hydrogel film		Rat (abdominal)	[458]
	Polylactide-PEG tri-block copolymer (PELA)		Mouse, Rat (abdominal)	[459,460]
	Poloxamer 407 (PEG-polypropylene glycol-PEG)	FloGel (Alliance Pharm Co.)	Rat, Hamster, Rabbit (peritoneal)	[428,461,462,463]
	PEG-poly(α-hydroxy acid) diacrylate macromers		Rat (peritoneal), Rabbit (pelvic)	[464]
	PEG-chitostan		Rat (peritoneal)	[465]
	PEG-poly(ε-caprolactone)-PEG (PECE) hydrogel		Mouse, Rat (peritoneal)	[466]
	Silicone		Canine (peritoneal)	[467]
	Polyethylene		Rat (peritoneal)	[45]
	**Poly(vinyl alcohol) (PVA)**		Rabbit (peritoneal)	[468]
			Rat (pericardial)	[469]
	PVA-gelatin membrane		Rat (peritoneal)	[470]
	PVA hydrogel		Porcine, Rat, Human (peritoneal)	[471,472,473]
			Rabbit (abdominal)	[474]
	PVA-coated polyester mesh		Rat (peritoneal)	[475]
	PVA-CMC hydrogel		Rabbit (abdominal)	[476]
	PVA-CMC trilaminar membrane		Rabbit (pericardial)	[477]
	**Polylactic acid (PLA)**		Rat (peritoneal)	[270,335,465,478]
			Rabbit, Porcine (pericardial)	[314,479]
	PLA nanosheets		Mouse (peritoneal)	[480]
	PLA gel		Human (skin-flap)	[481]
	PLA-PEG copolymer membrane		Rat (Peritoneal, pericardial)	[482]
			Canine (pericardial)	[483]
	PLA-PEG-ePTFE bioresorbable polymer		Rabbit (pericardial)	[484]
	**Poly ε-caprolactone (PCLA)**		Rat (abdominal)	[485,486]
	Hyaluronic acid-loaded poly(ε-caprolactone)		Rat (abominal)	[487]
	Poly(ε-caprolactone-co-lactide)-bpoly(ethyleneglycol)-b-poly(ε-caprolactoneco-lactide)(PCLA-PEG-PCLA)		Rabbit (peritoneal)	[488]
	Poly(ethylene glycol)-poly (ε-caprolactone)-poly(ethylene glycol) (PEG-PCLA-PEG, PECE)		Rat (peritoneal)	[489]
	Poly(ε-caprolactone)-poly(ethylene glycol)-poly(ε-caprolactone) (PCLA-PEG-PCLA) micelles		Rat (peritoneal)	[490]
	**Poly-β-hydroxybutyrate**		Bovine, Ovine (pericardial)	[491,492]
	PEG-poly-β-hydroxybutyrate valerate		Rat (peritoneal)	[465]
**Composite Polymers**				
	Polyethylene oxide-sodium carboxymethylcellulose gel	Oxiplex/AP Gel (FzioMed) Intercoat (Ethicon)	Human (pelvic)	[493,494,495,496]
			Rat (peritoneal)	[497]
	Silicone-urethane-polyester copolymer	(UBE Sheet, UBE Industries Ltd.)	Canine (pericardial)	[498]
	HA-3,3′ -dithiopropionic dihydrazide/PEG diacrylate (HA-DTPH/PEGDA)	Carbylan-SX (Carbylan Biosurgery Inc.)	Rat (peritoneal), Rabbit (pericardial)	[499]
			Rabbit (pericardial)	[500]
	Poloxamer-alginate-CaCl2 (gel)		Rabbit (pericardial)	[501]
	Carboxymethyldextranhydrazide-DX-aldehyde		Rabbit (peritoneal)	[427]
	Poly(lactic acid)-poly(oxyethylene-cooxypropylene) (PLA-Pluronic F68)		Rat (peritoneal)	[337]
	d,l-polylactide-ε-caprolactonetrimethylenecarbonate (PCT co-polymer)		Rat (peritoneal)	[370]
	Monomethoxy poly(ethylene glycol)-poly(lactic acid) hydrogel		Rat (abdominal)	[502]
	PLGA-poly(lactide-co-caprolactone) (PLCA)-poly(L-phenylalanine-co-p-dioxanone (PDPA) film		Rabbit (abdominal)	[503]
	Oxidized regenerated cellulose-polypropylene-poly(ε-caprolactone)		Rat (abdominal)	[504]
				
**Drug-Loaded Composite Polymers**				
**Antibiotics**				
	Cefoxitin sodium in PLGA/PEG-PLA membrane (nanofibrous sheet)		Rat (peritoneal)	[412]
	Ornidazole in PCL (nanofibrous sheet)		Rat (peritoneal)	[486]
	Chloramphenicol in dextran (solution)		Rat (peritoneal)	[505]
**Anti-coagulant (Heparin)**				
	Heparin in Interceed		Rabbit, human (peritoneal)	[240,506]
	Heparin in Seprafilm		Rat (peritoneal)	[507]
	Heparin in collagen		Canine (peritoneal)	[508]
**Anti-inflammatory**				
	Ibuprofen in PLLA-PEG copolymer (dense or nanofibrous sheet)		Rat (peritoneal)	[410]
	Ibuprofen in pluronic mixtures-crosslinked ALG (hydrogel)		Rat (peritoneal)	[428]
	Ibuprofen in poly(lactic-co-glycolic acid) (nanofibrous mesh)		Mouse (abdominal)	[509]
	Ibuprofen in PVA cryobarrier		Rat (abdominal)	[510]
	Dexamethasone in poly(lactide-co-glycolide) microparticles		Rat (peritoneal)	[511]
	Dexamethasone in PLA-PEG copolymer		Rabbit (pericardial)	[512]
	Budesonide in cross-linking HA (hydrogel)		Rabbit (abdominal)	[513]
	Tolmetin in HA		Rat (abdominal)	[170]
	Naproxen nanoparticles in chitosan hydrogel		Rat (abdominal)	[417]
	Atorvasain in Seprafilm (sheet)		Rat (peritoneal)	[514]
	Vitamin E/Seprafilm (sheet)		Rat (peritoneal)	[515]
	Methylene blue in polyhydroxybutyrate (nanofiber sheet)		Rat (abdominal)	[174]
**Fibrinolytic Agents**				
	tPA in Interceed		Rat (peritoneal)	[516]
	tPA in HA-adipic dihydrazide-HA-aldehyde (hydrogel)		Rabbit (peritoneal)	[517]
	Tranilast-encapsulated polydioxanone fiber in CMC (hydrogel)		Rabbit (peritoneal)	[518]
	Streptokinase in polyhydroxybutyrate-co-hydroxyvalerate membrane		Rat (peritoneal)	[215]
**Anti-Cancer and Anti-Tumour Drugs**				
	Paclitaxel in crosslinked HA		Rat (peritoneal)	[519]
	Doxorubicin in poly(ethylene glycol)-poly(ε-caprolactone)-poly(ethylene glycol) (PECE) copolymer		Mouse (peritoneal)	[520]
	Mitomycin C in crosslinked HA (hydrogel)		Rat (abdominal)	[225]
	Rapamycin in poly(ε-caprolactone)-poly(ethylene glycol)-poly(ε-caprolactone) (PCL-PEG-PCL) (sheet)		Porcine (pericardial)	[521]
**Growth Factors**				
	Keratinocyte growth factor (KGF) in N,O-carboxymethyl chitosan (hydrogel)		Rat (abdominal)	[522]
	Epidermal growth factor (EGF) in gelatin (sheet)		Porcine (pericardial)	[523]
**Other Drugs**				
	Tranilast in sodium carboxymethylcellulose (sheet)		Rabbit (abdominal, pelvic)	[524]
	Ginsenoside Rg1 in acellular bovine pericardium		Rabbit (pericardium)	[525]
	Fibrin in agarose hydrogel patch		Rat (abdominal)	[526]

## 6. Perspective

Post-surgical adhesions pose a great health and financial burden. A substantial amount of work has been dedicated to better understanding the exact mechanism of adhesion formation in the surgical setting. Inflammation and immune mediators have long thought to play a central role in the development and severity of post-operative adhesions. Although our knowledge of some of the underlying mechanisms driving adhesion formation has significantly improved over the past two decades, literature has yet to fully explain the pathogenesis and etiology of post-surgical adhesions. As a result, finding an ideal preventative strategy and leveraging appropriate tissue engineering strategies has proven to be difficult.

Primary prevention of post-operative adhesions involves careful and meticulous surgical work causing minimal tissue injury. Some groups believe that this can also be accomplished via laparoscopic surgery as an alternative to open surgery [527,528]. However, for select surgeries, there is no substitute for open surgery. Preventing post-surgical adhesion formation is difficult for two main reasons. First, adhesions form as a result of a coordinated physiologic response to tissue injury. Second, the exact mechanisms driving adhesion formation are not well understood. Therefore, preventative measures that have been proposed to date have not been completely and consistently successful once translated into clinical settings. In some cases, they have led to serious adverse events, including infection and irreversible disruption of the physiologic healing process. Unfortunately, experimental animal models often produce results that are far superior to those seen in human clinical trials, rendering preclinical results not reproducible. For these reasons, although a large number of preventative and therapeutic options have been developed, their clinical translation has generally been limited.

As reviewed here, pharmaceutical agents and different barrier strategies have been used to varying degrees of success in preventing or minimizing post-operative adhesions. Drugs that target either inflammatory pathways or components of the clotting cascade have been used, but their clinical success has been limited due to systemic effects or inhibition of normal healing processes. Barriers, which range from natural to synthetic to a mixture of both, have been designed to create frictionless surfaces between tissue layers. The clinical application of these products has been restricted for different reasons, such as requiring a dry, bloodless surface and challenges with placing a barrier in irregularly-shaped areas.

The development of successful biomaterials for post-surgical adhesion will likely need to employ an understanding of the surface characteristics of these biomaterials in addition to a deepened understanding of the physiological mechanisms behind adhesion formation. Future biomaterials will likely have multiple favorable characteristics through the use of a mixture of natural and synthetic polymers, copolymers, use of novel preparation methods, and by leveraging the drug loading capabilities of biomaterials. The design of pharmaceuticals and barriers will have to focus on the promotion of wound healing, hemostatic, and even regenerative capacities of biomaterials, while leveraging anti-adhesive properties of currently established barrier methods [7]. Multifunctional or multi-layer biomaterials that can employ a variety of anti-adhesive and regenerative strategies with different structural compositions can build on the advantages of copolymers. These strategies will set the foundation for the design of systems that can incorporate stem cells to promote and induce tissue regeneration. Moreover, further customization for each tissue cavity can be possible, leading to the restoration of tissue function, promoting fibrinolysis, and reducing friction through lubrication [529]. It is also feasible to augment these multilayered structures with multiple drugs to leverage their effects on specific components of adhesion formation pathways, or the incorporation of immune moieties for the regulation of the immune system to promote healing [529]. The role of nanoparticles and gene therapy in preventing post-surgical adhesions should also be meticulously assessed. These emerging strategies are promising in theory and potentially offer an ideal platform for facilitating precision and personalized medicine. Ultimately, any potential strategy must be safe, effective, user-friendly, have a readily-accessible delivery method, and ideally, be biodegradable. Furthermore, more robust basic and pre-clinical studies are needed. In addition to considering different tissue planes and cavities, such studies should carefully assess the safety and efficacy of preventative measures for various types of surgeries and the delivery options available to surgeons. Finally, to establish clinical relevance, current and future agents must be evaluated in rigorous, large, multicenter, randomized, controlled trials that include patients undergoing different types of surgery.

## 7. Conclusions

Post-surgical adhesions are fibrotic connections that form between organ surfaces and the walls of surrounding body cavities following tissue trauma and ischemia. To varying degrees of success, different strategies have been employed to mitigate the challenges associated with post-surgical adhesions. Herein, we have summarized established methods and products that have been used in pre-clinical and clinical studies. We have also examined emerging approaches that have the potential to facilitate precision medicine in mitigating post-surgical adhesions.

## Figures and Tables

**Table 1:** Cellular and signalling pathways involved in adhesion formation.

Factor	Component	Role in Adhesion Formation	Reference
Surgical Trauma		Increase of fibrin Increase levels of plasminogen activator inhibitor Induction of local inflammatory response Hypoxia and reactive oxygen species (ROS) release leading to inflammation and activation of coagulation cascade Surgical hypoxia may decrease fibrinolysis	[16,23,26,27,28,30,31,32,33,34]
Extracellular Matrix Components	Fibronectin, hyaluronic acid, glycosaminoglycans, proteoglycans	Matrix for proliferation of cellular components Secreted by fibroblasts	[35,36]
Cellular Mediators			[37,38]
	Fibroblasts and Myofibroblasts	Subperitoneal fibroblast deposition required for adhesion development Transition to myofibroblast phenotype associated with long-lasting adhesions Maturation of adhesions through collagen and extracellular matrix (ECM) production	[39,40,41,42,43,44,45,46,47,48]
	Mesothelial Cells	Potential protective role Insult induces pro-fibrotic phenotype and secretion of inflammatory mediators, cells, and ECM components that contribute to immune cell recruitment and coagulation Mesothelial to mesenchymal transition (MMT) drives adhesion formation	[49,50,51,52,53]
	Macrophages	Identified in long-lasting adhesions Fundamental in adhesion formation Secrete fibrinolytic mediators and interleukins Recruit and influence mesothelial cells	[54,55,56,57,58,59]
	Neutrophils	Recruited by activated mesothelial cells Release ROS, inhibiting fibrinolysis and exerting a direct cytotoxic effect on mesothelial cells Debated in the literature to have both a pro- and anti-adhesive effect	[50,60,61,62,63,64]
	T Lymphocytes	Persist in quality and quantity in long-lasting adhesions Th1, Th2, and Treg CD4+ phenotypes implicated in adhesion formation Produce pro-inflammatory cytokines	[54,65,66,67]
	Mast Cells	High concentrations in post-surgical adhesions Release histamines, serotonin, cytokines, serine proteases, vascular endothelial growth factor (VEGF), and chymase Deficiencies in mast cells reduce adhesion formation	[68,69,70]
Signalling Factors			
	Coagulation Cascade	Production of thrombin, key activator of fibrin	[23]
	Fibrin-Fibrinolysis Balance	Disruption of balance between fibrin production and fibrinolysis leads to adhesion formation Dysregulation between plasminogen-plasmin, and plasminogen activator inhibitors (PAIs) Fibrin matrix allows fibroblast adhesion and ECM maturation	[71,72]
	Matrix Metalloproteinases	Post-surgical shifts in ratios of matrix metalloproteineases (MMPs) to tissue inhibitors of MMPs (TIMPs) MMP-2/9 proposed as markers for adhesion formation Chronic suppression of MMP/TIMP ratios lead to adhesions	[73,74,75,76,77]
	Interleukins	High concentrations in adhesion sites and some direct correlations to extend of adhesion formation Pro-inflammatory effects Increased recruitment of immune cells	[38,78,79,80,81,82,83,84,85,86]
	TNF-α	Abundant in peritoneal fluid post-surgery Increases interleukin production	[87,88]
	TGF-β	Key fibrotic mediator Elevated in adhesions Stimulates myofibroblast migration and activation Chemotactic for neutrophils, T-cells, monocytes, and fibroblasts Induces ECM production Inhibits matrix degradation by altering ratio of protease to protease inhibitors	[82,89,90,91,92,93,94]
	VEGF	Promotes angiogenesis, involved in coagulation and fibrinolysis Increases vascular permeability and promotes fibrin matrix deposition	[95,96,97]

**Table 2:** Materials commonly used in adhesion prevention and a brief description of possible reasons for efficacy in adhesion prevention. Adapted from [10].

Material	Reasoning for Efficacy
Polytetrafluoroethylene (PTFE)	Physiologically inert Low adhesiveness with cells/tissues Separates damaged surfaces during wound healing without degradation Biocompatible
Polylactic acid (PLA)	No specific binding site with cells/tissues on polymer matrix Low adhesiveness with cells/tissues Separates damaged surfaces during wound healing without degradation Biocompatible (FDA approval for human use in orthopedic and neurosurgical operations) Biodegradable
Polyethylene glycol (PEG)	High mobility and steric stabilization effects in aqueous solution Low adhesiveness with cells/tissues Biocompatible
PLA-PEG	Low adhesiveness with cells/tissues Flexible and hydrophilic Biocompatible Biodegradable
Hyaluronic Acid (HA)	Wound healing properties High viscosity when dissolving by water or body fluid Muco-adhesive property in solid state Biocompatible Bioresorbable
Alginate (ALG)	Wound healing properties Muco-adhesive property in solid state Partially crosslinking by multi-valence positive charged ions in body fluid Biocompatible Bioresorbable
Cellulose (oxidized regenerated) (ORC)	Wound healing properties, in terms of re-epithelialization Muco-adhesive property in solid state Biocompatible Bioresorbable
Carboxymethyl cellulose (CMC)	Remained on the injury surfaces during wound healing Muco-adhesive property in solid state Delayed bioresorption Biocompatible
Icodextrin	Metabolized into oligosaccharides by the α-amylase in the body Delayed bioresorption in peritoneal cavity Remained on the injury surfaces during wound healing Biocompatible
